# Tolerogenic nanoparticles mitigate the formation of anti-drug antibodies against pegylated uricase in patients with hyperuricemia

**DOI:** 10.1038/s41467-021-27945-7

**Published:** 2022-01-12

**Authors:** Earl Sands, Alan Kivitz, Wesley DeHaan, Sheldon S. Leung, Lloyd Johnston, Takashi Kei Kishimoto

**Affiliations:** 1 Selecta Biosciences, Watertown, MA USA; 2 Altoona Center for Clinical Research, Altoona, PA USA; 3 Present Address: Parexel International, Waltham, MA USA

**Keywords:** Inflammatory diseases, Translational research, Antibodies, Phase I trials

## Abstract

Biologic drugs have transformed the standard of care for many diseases. However, many biologics induce the formation of anti-drug antibodies (ADAs), which can compromise their safety and efficacy. Preclinical studies demonstrate that biodegradable nanoparticles-encapsulating rapamycin (ImmTOR), but not free rapamycin, mitigate the immunogenicity of co-administered biologic drugs. Here we report the outcomes from two clinical trials for ImmTOR. In the first ascending dose, open-label study (NCT02464605), pegadricase, an immunogenic, pegylated uricase enzyme derived from *Candida utilis*, is assessed for safety and tolerability (primary endpoint) as well as activity and immunogenicity (secondary endpoint); in the second single ascending dose Phase 1b trial (NCT02648269) composed of both a double-blind and open-label parts, we evaluate the safety of ImmTOR (primary endpoint) and its ability to prevent the formation of anti-drug antibodies against pegadricase and enhance its pharmacodynamic activity (secondary endpoint) in patients with hyperuricemia. The combination of ImmTOR and pegadricase is well tolerated. ImmTOR inhibits the development of uricase-specific ADAs in a dose-dependent manner, thus enabling sustained enzyme activity and reduction in serum uric acid levels. ImmTOR may thus represent a feasible approach for preventing the formation of ADAs to a broad range of immunogenic biologic therapies.

## Abstract (web summary)

Anti-drug antibodies (ADA) induced by biologic drugs may hamper the efficacy of treatment, so inhibiting ADA induction is desirable. Here, in two clinical trials, the authors show that ImmTOR, previously reported to reduce drug immunogenicity in animal studies, helps mitigate ADA induced by pegylated uricase for treating patients with hyperuricemia.

## Introduction

Pegylated uricases, such as pegloticase and pegadricase, are enzyme therapies that rapidly metabolize uric acid and are promising therapies for the treatment of chronic gout in adult patients that are refractory to conventional therapy^1–5^. The therapeutic goal in gout is to reduce serum uric acid levels (sUA) below 6 mg/dL, as uric acid can crystalize at higher levels and form monosodium urate (MSU) crystals in joints and soft tissues that cause episodes of gout flares, remodeling of bone, and debilitating pain^6^. Available oral therapies cannot efficiently eliminate large MSU crystal deposits, called tophi, as sUA levels must be maintained well below 6 mg/dL to drive the dissolution of urate crystals^1^. In contrast, pegylated uricase can reduce sUA levels below 2 mg/dL resulting in accelerated resolution of tissue MSU deposits^5^. However, pegylated uricases are highly immunogenic resulting in the formation of anti-drug antibodies (ADAs) in ~90% of patients^2,7^, correlating with the loss of therapeutic benefit and increased infusion reactions^2^.

**Fig. 1:**
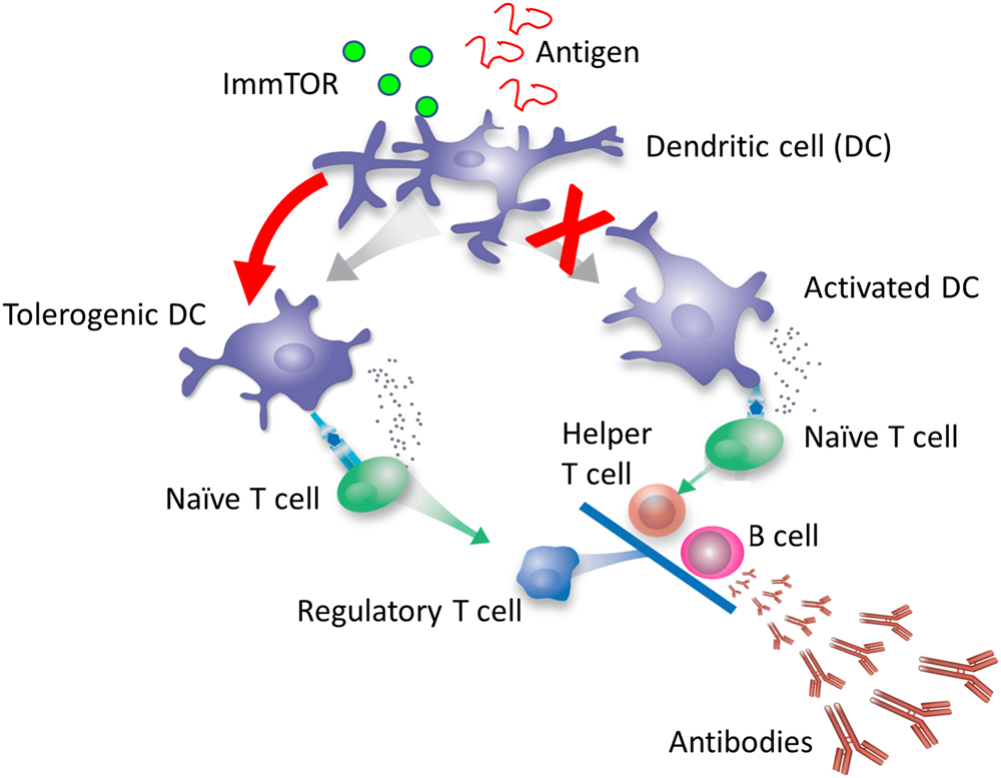
Schematic of ImmTOR mechanism of action. ImmTOR is selectively taken up by antigen-presenting cells, such as dendritic cells (DC) in the spleen and liver. ImmTOR induces tolerogenic DCs that process and present co-administered antigen in a manner that results in the expansion of antigen-specific regulatory T cells. The Tregs inhibit activation of effector T cells and prevent the formation of anti-drug antibodies. Adapted from Kishimoto^8^.

We have developed ImmTOR nanoparticles encapsulating rapamycin, an inhibitor of the mTOR pathway, to induce selective immune tolerance to co-administered biologic drugs^8,9^. Rapamycin inhibits effector T cell activation and is used clinically as part of a chronic immunosuppressive regimen to prevent renal allograft rejection^10^. However, in vitro studies have shown that rapamycin can also induce tolerogenic antigen presenting cells (APCs) which promote activation of regulatory T cells^11^. ImmTOR nanoparticles show selective biodistribution to the spleen and liver following intravenous administration where they are taken up by antigen-presenting cells (APCs)^9,12–14^. ImmTOR induces a tolerogenic phenotype in dendritic cells in the spleen^9^ and in liver sinusoidal endothelial cells and other APCs in the liver^12^ (Fig. 1). Co-administration of ImmTOR with antigen results in the induction or expansion of antigen-specific regulatory T cells (Tregs)^9,13,15^. Induction of specific immune tolerance by ImmTOR is supported by the following evidence: (1) ImmTOR induces Tregs specific to the co-administered antigen^9,13,15^; (2) tolerance can be transferred by adoptive transfer of splenocytes from treated animals to naïve recipients^14–16^; (3) the tolerance is durable to subsequent challenge with antigen alone^9,13,14^; and (4) animals tolerized to a specific antigen are capable of responding to an unrelated antigen^9,13,14,16^. Preclinical studies showed the ability of ImmTOR to mitigate immunogenicity of pegadricase resulting in sustained control of sUA in uricase-deficient mice^9^.

Here we evaluate the ability of ImmTOR to prevent the development of ADAs against pegadricase in patients with hyperuricemia. The addition of ImmTOR to pegadricase results in a dose-dependent reduction in ADAs corresponding with prolonged serum enzyme activity and maintenance of low sUA levels. These results suggest that ImmTOR could enable monthly dosing of pegadricase in patients with uncontrolled gout, and also implicate the combination of ImmTOR with other biologic therapies to mitigate adverse effects of immunogenicity.

## Results and discussion

**Fig. 2:**
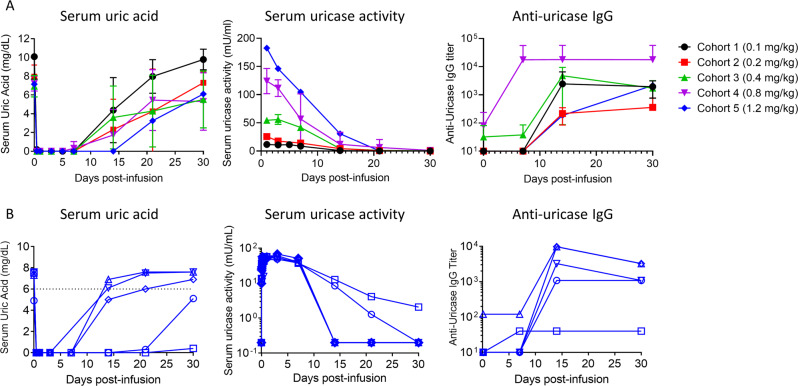
Single ascending dose study of pegadricase in patients with hyperuricemia. **A** Patients with sUA > 6 mg/dL at screening were assigned to one of five cohorts receiving a single IV infusion of pegadricase (0.1, 0.2, 0.4, 0.8 or 1.2 mg/kg). Each cohort consisted of 5 patients, except for cohort 5 (1.2 mg/kg) which enrolled 2 patients. Blood was drawn at various timepoints indicated and sUA levels were determined. Lines represent the mean for each cohort and error bars represent the standard deviation. Only the mean is depicted for cohort 5. **B** Serum uric acid, serum pegadricase activity, and anti-uricase IgG antibodies are shown for the five individual patients in cohort 3 (0.4 mg/kg pegadricase). One patient (square symbols) developed only low anti-uricase IgG titers and showed prolonged reduction of sUA and sustained serum pegadricase activity. Source data are provided as a Source Data file.

Patients with hyperuricemia were screened at baseline for sUA > 6 mg/dL (Supplementary Tables 1 and 2) and administered escalating doses of pegadricase (without ImmTOR) in a Phase 1a multicenter, sequential, single-ascending dose, open-label study (NCT02464605). The primary endpoint was to assess the safety and tolerability of of pegadricase, and the secondary endpoints included evaluation of the activity and immunogenicity of pegadricase. Twenty-two patients were enrolled in the study and were assigned to one of five cohorts receiving a single IV infusion of pegadricase (0.1, 0.2, 0.4, 0.8, or 1.2 mg/kg) (Supplementary Figure 1). Pegadricase was well tolerated at all doses tested (Supplementary Table 3). Intravenous dosing of pegadricase resulted in a rapid and dramatic drop in sUA levels in all subjects, but sUA levels returned to baseline between 7 and 30 days after dosing in most patients at all dose levels tested (Fig. 2A). As expected, pegadricase was highly immunogenic. All subjects developed anti-uricase IgG antibodies by day 30 after a single dose of pegadricase. Serum uric acid data for each subject in Cohort 3, dosed with 0.4 mg/kg pegadricase, are shown in Fig. 2B. The sUA levels returned to baseline by day 30 in 4 of 5 patients, correlating with the development of anti-uricase IgG antibody titers >1000 by 14 days after dosing and the loss of serum enzyme activity (Fig. 2B). However, one patient (open squares) dosed with 0.4 mg/kg pegadricase developed only a low titer of 1:120 and was able to maintain serum enzyme activity and low sUA levels for at least 30 days after a single dose (Fig. 2B). We hypothesized that a dose of 0.4 mg/kg pegadricase would be able to support a monthly treatment regimen if the formation of ADAs could be mitigated.

**Fig. 3:**
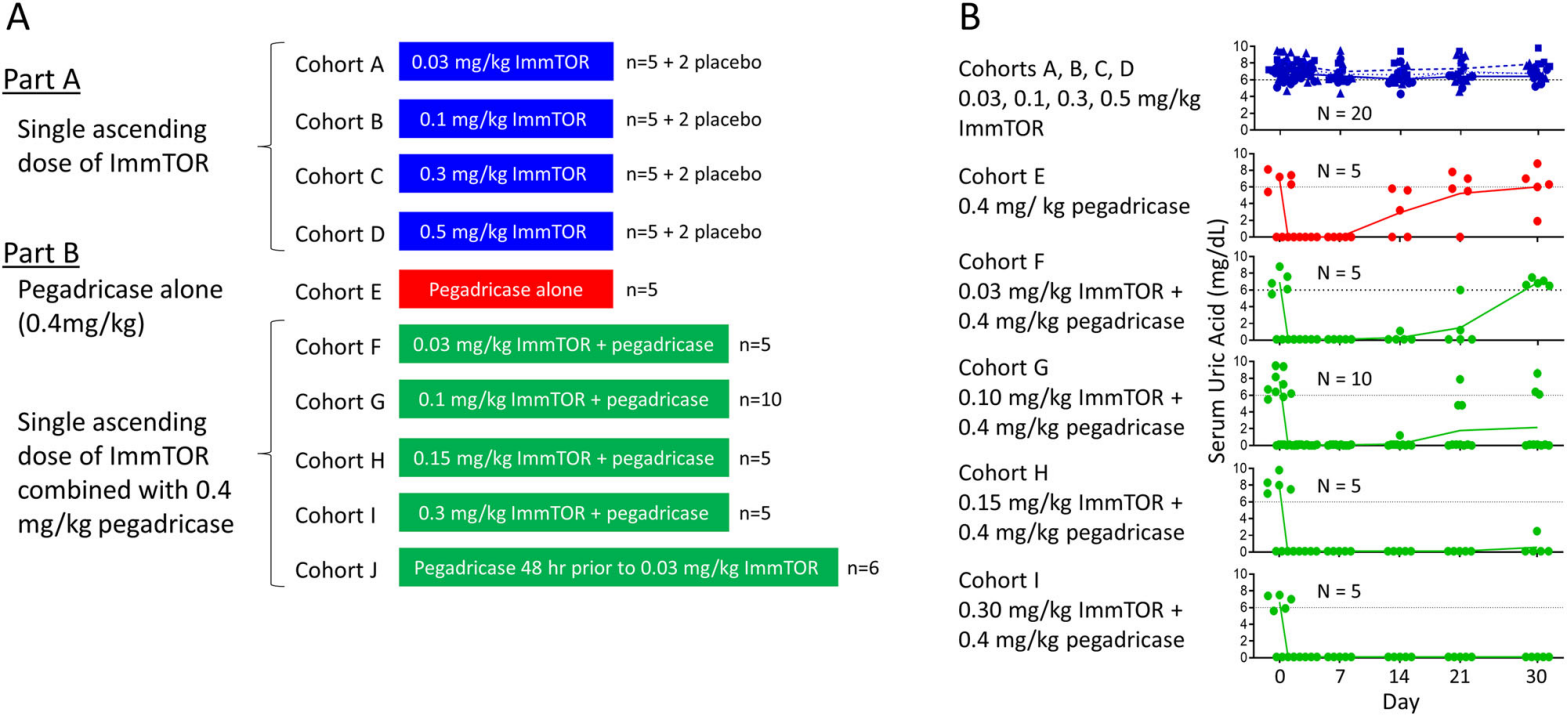
Single ascending dose study of ImmTOR and SEL-212 in patients with hyperuricemia. **A** Schematic of cohorts dosed with pegadricase alone (Cohort A), single escalating doses of ImmTOR alone (Cohorts B–E), or single escalating doses of SEL-212 (Cohorts F–J, 0.03, 0.1, 0.15, or 0.3 mg/kg ImmTOR combined with a fixed dose of 0.4 mg/kg pegadricase). Cohorts B–E were double blinded and randomized to either placebo or study drug in a 2:5 rato, while Cohorts A and F–J were open-label. All cohorts consisted of 5 drug-treated patients, except for Cohort G, that contained 10 patients and Cohort J which contained 6 patients. In addition, Cohorts B–F each contained two patients that received placebo (vehicle). Patients in Cohorts F–I received an IV infusion of ImmTOR followed immediately by an IV infusion of pegadricase. Patients in Cohort J received an IV infusion of pegadricase followed 48 h later by an IV infusion of ImmTOR. **B** Serum uric acid levels over time. Each symbol represents sUA levels for an individual patient at various timepoints indicated and the lines indicate mean sUA levels for the cohort. The dotted black line represents 6 mg/dL sUA. Source data are provided as a Source Data file.

The 0.4 mg/kg dose of pegadricase was taken into the next study to be evaluated in combination with ImmTOR. Study SEL-212/101 ((NCT02648269) was a Phase 1b multi-center clinical trial conducted in two parts. Part A was a double-blind, placebo-controlled, sequential, single-ascending-dose safety study of ImmTOR, and Part B was an open-label, sequential, single-ascending dose study of SEL-212, a combination product candidate consisting of a fixed dose of pegadricase combined with ascending doses of ImmTOR, in patients with hyperuricemia with or without a clinical diagnosis of gout (Fig. 3A). The primary endpoint was the safety and tolerability of ImmTOR and SEL-212. Additionally, the study was designed to assess secondary endpoints of the activity and immunogenicity of pegadricase when dosed in combination with ImmTOR. In total, 64 patients were enrolled (Supplementary Tables 1 and 4) and 62 patients completed the study (Supplementary Figs. 2A and 2B).

Cohorts in Part A treated with 0.03, 0.1, 0.3, and 0.5 mg/kg ImmTOR (in the absence of pegadricase) showed no change in sUA levels, as expected (blue symbols, Fig. 3B). The ImmTOR was well tolerated at doses up to 0.3 mg/kg, and the majority of treatment-emergent adverse events (TEAEs) were mild or moderate in severity and most were not related to study drug (Table 1 and Supplementary Table 5). At 0.5 mg/kg, the highest dose tested, two subjects experienced serious adverse events (SAEs) of stomatitis, a known side-effect of rapamycin^17^. As a result, the ImmTOR dose was limited to 0.3 mg/kg in Part B of the study involving combination treatment arms.

**Table 1:** Phase 1b Treatment Emergent Adverse Events.

	Placebo	ImmTOR				Pegadricase	SEL-212				SEL-212*
ImmTOR (mg/kg)	0	0.03	0.1	0.3	0.5	0	0.03	0.1	0.15	0.3	0.03
Pegadricase (mg/kg)	0	0	0	0	0	0.4	0.4	0.4	0.4	0.4	0.4
*N*	8	5	5	5	5	5	5	10	5	5	6
Subjects with at least one											
TEAE, *n* (%)	2 (25.0)	3 (60.0)	4 (80.0)	4 (80.0)	5 (100)	3 (60.0)	2 (40.0)	9 (90.0)	3 (60.0)	5 (100)	2 (33.3)
Severe TEAE, *n* (%)	0	0	0	0	2 (40.0)	0	0	0	1 (20.0)	0	0
Life-Threatening TEAE, *n* (%)	0	0	0	0	0	0	0	0	0	0	0
Drug-related TEAE, *n* (%)	1 (12.5)	2 (40.0)	2 (40.0)	3 (60.0)	5 (100)	1 (20.0)	1 (20.0)	5 (50.0)	2 (40.0)	3 (60.0)	1 (16.7)
Drug-related serious TEAE, *n* (%)	0	0	0	0	2 (40.0)	0	0	2 (20.0)	0	0	0
Deaths, *n* (%)	0	0	0	0	0	0	0	0	0	0	0
TEAE leading to discontinuation of study drug, *n* (%)	0	0	1 (20.0)	0	0	0	0	0	0	0	1 (16.7)

*Cohort was dosed with pegadricase followed 48 hrs later by ImmTOR. All other SEL-212 cohorts were dosed with ImmTOR first followed immediatedly by pegadricase.

**Fig. 4:**
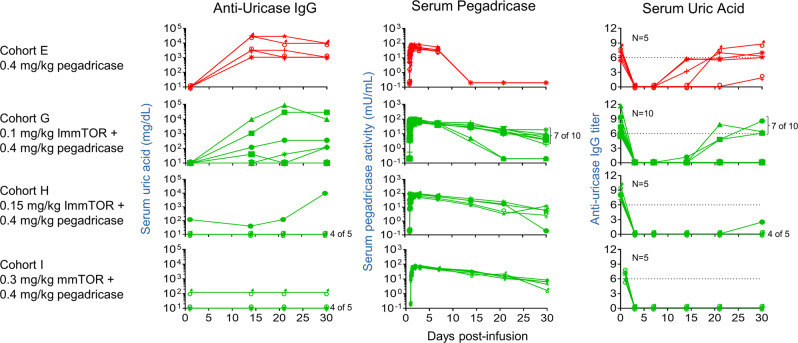
Correlation of sUA with anti-uricase IgG and serum pegadricase activity. Anti-uricase IgG titers, serum pegadricase activity, and sUA are plotted against time for individual patients in cohorts A, G, H, and I. Each line represents an individual patient.

A control cohort of patients treated with 0.4 mg/kg pegadricase alone, mimicking Cohort 3 from the Phase 1a study, showed a transient drop in sUA for 7 days that rebounded back to baseline levels by day 30 in four of five patients (red symbols, Fig. 3B), similar to that observed in the Phase 1a study (Fig. 2B). The addition of escalating doses of ImmTOR with a fixed dose of 0.4 mg/kg pegadricase showed a dose-dependent effect on sustained reduction in sUA levels (green symbols, Fig. 3b), with all patients maintaining sUA levels well below 6 mg/dL through Day 30 at ImmTOR doses of 0.15 and 0.3 mg/kg. The prolonged maintenance of low sUA levels correlated with the inhibition of anti-uricase IgG formation and maintenance of serum uricase activity (Fig. 4). Two patients showed low levels of pre-existing antibodies to uricase at baseline. Importantly, the patient dosed with 0.3 mg/kg ImmTOR and 0.4 mg/kg pegadricase showed no further increase in the levels of anti-uricase antibodies from baseline, although the patient dosed with 0.15 mg/kg ImmTOR and 0.4 mg/kg pegadricase developed ADAs by Day 30 (Fig. 4). In addition to the ADAs directed against the protein backbone of pegadricase, some patients developed antibodies against the PEG moiety. In general, the anti-PEG antibodies were transient and occurred only in a subset of patients that developed anti-uricase IgG. Importantly, ImmTOR treatment was also effective at inhibiting the anti-PEG antibody response (Supplemental Table 5).

**Fig. 5:**
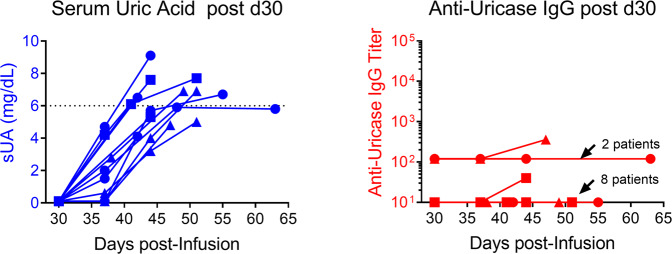
Serum uric acid and anti-uricase IgG for selected patients followed past day 30. Patients in cohorts G, H, and I that maintained sUA levels below 6 mg/dL for 30 days were selected on a voluntary basis to participate in additional monitoring. Serum uric acid and anti-uricase IgG titers are plotted for individual patients at various timepoints indicated. Patients in cohorts G, H, and I are represented by circle, square, and triangle symbols, respectively. All patients showed a rebound in sUA levels by day 50, indicating clearance of enzyme. Ten of the twelve patients showed no increase in anti-uricase IgG titers after day 30 and the remaining two patients showed only modest elevations in anti-uricase IgG titers. Source data are provided as a Source Data file.

Patients dosed with 0.1, 0.15, and 0.3 mg/kg ImmTOR and pegadricase were invited to return for additional follow-up visits on a voluntary basis. In those patients who showed sUA levels <6 mg/dL at day 30, serum uric acid levels gradually returned to baseline levels by day 51 after a single dose of SEL-212 (Fig. 5). Importantly, anti-uricase antibodies did not emerge or substantially increase during this interval (Fig. 5), suggesting that the return of sUA to baseline levels was due to metabolism and clearance of the enzyme and not to delayed formation of ADAs.

A single IV infusion of SEL-212 (ImmTOR plus pegadricase) was well tolerated (Table 1 and Supplemental Tables 6 and 7). No deaths or life-threatening treatment-emergent adverse events (TEAEs) were reported during the study, and overall, there were no notable trends in the nature or frequency of TEAEs. Two subjects that received 0.1 mg/kg ImmTOR in combination with 0.4 mg/kg pegadricase were reported with study drug-related serious adverse events (SAEs) of moderate severity. One subject experienced a drug hypersensitivity rash and the other subject experienced acute kidney injury and pneumonia. Both subjects recovered from the events. No drug-related SAEs were observed at the higher doses of 0.15 or 0.3 mg/kg ImmTOR combined with pegadricase.

ADAs are a significant barrier to clinical use of pegylated uricase^18^. Pegloticase, a marketed version of a pegylated uricase approved for the treatment of chronic gout in adult patients refractory to conventional therapy, induced ADAs in 92% of patients in Phase 3 clinical trials, which affected both efficacy and safety^2,7,18^. Only 42% and 35% of patients showed durable responses with biweekly and monthly dosing, respectively^2^. The loss of efficacy was associated with the development of high titer ADAs. Only the biweekly regimen was approved for marketing; however, the monthly dosing cohorts are informative, showing that the effects of ADAs on efficacy can be seen at 4 weeks after the first dose. Our data suggests that pegadricase may be even more immunogenic than pegloticase after a single dose. Overall, only 8 of 24 (33%) subjects administered a single dose of pegloticase (0.5–12 mg) developed ADAs, with no apparent dose response^19^. At the clinically efficacious dose of 8 mg, 0 of 4 subjects developed ADAs against pegloticase by 35 days after a single dose. In contrast, all subjects that were administered a single dose of 0.1, 0.2, or 0.4 mg/kg pegadricase developed ADAs by 30 days. However, the co-administration of pegadricase with ImmTOR inhibited the formation of ADAs in a dose-dependent manner and enabled prolonged control of sUA levels that would be consistent with the ability to administer monthly dosing.

Immunogenicity of biologics is a vexing problem for the biopharmaceutical industry, in some cases resulting in loss of promising therapeutic candidates in clinical development^20–24^. The Food and Drug Administration (FDA) has advocated that companies take a proactive risk-based approach to reduce and mitigate unwanted immunogenicity and, in some cases, have required post-marketing commitments to evaluate immune tolerance strategies^25,26^. Prevention of ADAs would not only provide better outcomes for patients but also has the potential to improve existing drugs or enable novel applications, such as re-dosing of adeno-associated viral (AAV) gene therapy^16^. The use of ImmTOR as an adjunct to biologic therapies offers a promising approach to minimize the healthcare and economic burden of ADAs. Preclinical studies have demonstrated the ability of ImmTOR to mitigate the formation of ADAs against a wide variety of biologic therapies, including adalimumab in a model of inflammatory arthritis^9^, coagulation factor VIII in a model of hemophilia A^27^, alglucosidase alfa in a model of Pompe disease^28^, recombinant immunotoxin in a model of mesothelioma^14^, and AAV gene therapy vectors^16,29^.

In conclusion, the outcomes from two clinical trials are reported here for the first time. ImmTOR was well-tolerated and showed a dose dependent inhibition of uricase-specific ADAs enabling sustained enzyme activity and reduction in serum uric acid levels (sUA) in patients with hyperuricemia. ImmTOR technology represents a novel approach to preventing the formation of ADAs to a broad range of immunogenic biologic therapies.

## Methods

### Materials

ImmTOR. ImmTOR is a drug product consisting of a biodegradable polymeric nanoparticle consisting of poly[D,L-lactide] (PLA) and poly[D,L-lactide]-block-poly-[ethylene-glycol] (PLA-PEG) encapsulating rapamycin^9^. ImmTOR was manufactured under cGMP using a single-emulsion/solvent-evaporation process to form a suspension of nanoparticles, followed by sterile filling and storage as a frozen suspension at –20 °C. Briefly, the first manufacturing step was the production of an oil-in-water emulsion by homogenization. The oil-phase consisted of methylene chloride, PLA, PLA-PEG, rapamycin and surfactant and the water phase consists of buffer and surfactant. The nanoparticles were formed from the emulsion as organic droplets which were hardened by removal of the organic solvent (methylene chloride). The process of methylene chloride removal was initiated by diluting the emulsion with Evaporation Buffer (DPBS) in a stainless steel vessel that was mixed at low shear while the headspace was purged with a steady stream of air. As the methylene chloride evaporated, the polymeric matrix hardened into nanoparticles. The suspension was filtered with DPBS using tangential flow filtration (TFF) to remove any unencapsulated rapamycin and polymer, as well as to reduce the concentration of surfactant. The nanoparticles were retained by the TFF membrane and permeate was discharged to waste. After the diafiltration was completed, the suspension was prefiltered through a 0.2 µm filter to reduce bioburden and transferred into a single use bag. Prior to filling in vials, the nanoparticles were diluted to the desired concentration and then filled using a standard sterile liquid filling line. The filled vials were frozen at −20 °C and stored as a frozen suspension.

Pegadricase. Pegadricase, a recombinant urate oxidase from Candida utilis and modified with multiple 20,000 MW polyethylene glycol (PEG) moieties^30^, was manufactured under cGMP by 3SBio (Shenzen, China). Briefly, pegadricase was produced under cGMP by fermentation as a soluble, enzymatically active protein contained in the cytoplasm in E. coli in a 40 L bioreactor. The recombinant protein was isolated from the cell lysate and purified by hydrophobic interaction chromatography, hydroxyapatite chromatography, and anion exchange chromatography. The resultant bulk native uricase was then pegylated by the covalent attachment of 20 kD methoxy PEG (mPEG) moieties. Excess unreacted/unconjugated mPEG was removed by diafiltration against the drug substance final formulation buffer. The drug substance was sterile filtered using a 0.22 µm filter and stored frozen at −70 °C prior to filling. The final drug product was filled in vials, which were then lyophilized and stored at 2–8 °C. Reasonable requests for pegadricase for non-human use will be granted under a Materials Transfer Agreement.

### Clinical trials

The protocol and its amendments, the consent form, and other relevant study documentation were approved by the Copernicus Group (Durham, NC), a central Institutional Review Board (IRB) and by each study center before initiation of the study. These studies were conducted in accordance with the ethical principles that have their origin in the Declaration of Helsinki and that are consistent with Good Clinical Practices and applicable regulatory requirements. All patients provided written informed consent.

SEL-037/101 Phase 1a Trial (NCT02464605). SEL-037/101 was a multi-center, open label, sequential, single-ascending-dose study to assess the safety, tolerability, activity, and immunogenicity of pegadricase in patients with hyperuricemia (sUA > 6 mg/dL at screening). Patients were recruited at four U.S. clinical research centers (Duncansville, PA, Dallas, TX, Miami, FL, and Orlando, FL). The Phase 1a trial was initiated 01-May-2015 and completed 11-November-2015. The primary objective of this study was to assess the safety and tolerability of a single intravenous infusion of pegadricase (also known as SEL-037) in an ascending cohort dose escalation study. The secondary objectives were to assess the pharmacokinetics (PK), pharmacodynamics (PD) (ability to reduce circulating uric acid) and immunogenicity of pegadricase after a single intravenous dose. Seventy-three male and female patients, 21-70 years of age, were screened for sUA levels >6 mg/dL. Major inclusion and exclusion criteria are detailed in Supplementary Table 1. Twenty-five patients were planned to be enrolled, and 22 patients were actually enrolled in the study (Supplementary Figure 1). Patients with sUA levels >6 mg/dL at screening were assigned to one of five sequential cohorts receiving a single IV infusion of pegadricase (0.1, 0.2, 0.4, 0.8, or 1.2 mg/kg) over a 60 min period. Patient demographics by cohort are shown in Supplementary Table 2. A clinical review of sUA data from the 0.2, 0.4, and 0.8 mg/kg cohorts revealed that there was no further extension in the duration of uric acid lowering beyond the 0.2 mg/kg pegadricase dose, thus a decision was made to prematurely stop enrollment of subjects in the 1.2 mg/kg group. At the time the decision was made, 2 subjects had already been dosed with 1.2 mg/kg pegadricase. Sera were collected at baseline, 0.5 h, 1 h, 3 h, 5 h, 7 h, 10 h, 24 h, day 3, day 7, day 14, day 21, and day 30 for analysis of sUA and at baseline, day 7, day 14, and day 30 for analysis of ADAs.

SEL-212/101 Phase 1b Trial (NCT02648269). SEL-212/101 was a multi-center, double-blind, single-ascending-dose study of ImmTOR integrated with an open-label, single-ascending-dose study of SEL-212 (ascending doses of ImmTOR combined with a fixed 0.4 mg/kg dose of pegadricase) to assess the safety, tolerability, activity, and immunogenicity of SEL-212 in patients with hyperuricemia (SUA > 6 mg/dL at screening). Patients were recruited at eight U.S. clinical research centers (Little Rock, AR, Miami, FL, Orlando, FL, Baltimore, MD, Minneapolis, MN, Duncansville, PA, Anniston, AL, Lakewood, CO). The Phase 1b trial was initiated 21-December-2015 and completed 28-December-2016. Sixty-four patients were enrolled, and 62 completed the study. In Part A of the study, four cohorts of 7 patients received treatment with placebo or escalating doses of ImmTOR (0.03 mg/kg, 0.1 mg/kg, 0.3 mg/kg, 0.5 mg/kg; Supplementary Figure 2A). The 7 subjects in each cohort were blinded and randomized to ImmTOR (5 patients) or placebo (2 patients). Investigators were blinded, but the site pharmacist and the nurse administering the IV syringe infusion were not blinded. In part B of the study, which was open label, patients were assigned to receive a single IV infusion of pegadricase alone (0.4 mg/kg) alone, or SEL-212 (0.03, 0.1, 0.15, or 0.3 mg/kg ImmTOR combined with 0.4 mg/kg pegadricase; Supplementary Fig. 2B). There were 5 patients per cohort in Part B, with the exception of the 0.1 mg/kg ImmTOR + 0.4 mg/kg pegadricase group for which an additional 5 patients were added under a protocol revision to increase the N, as these patients showed a heterogenous effect of ImmTOR on anti-uricase IgG responses and maintenance of low sUA. In addition, a cohort of 6 patients was added to assess the effect of dosing 0.4 mg/kg pegadricase 48 h before dosing of 0.03 mg/kg ImmTOR. There was no obvious benefit of dosing pegadricase 48 h prior to dosing ImmTOR versus dosing sequentially on the same day, so this strategy was not further pursued. Patient demographics by cohort are detailed in Supplementary Table 4. ImmTOR was infused over a period of 55 min and pegadricase was infused over a period of 60 min. Those patients receiving the combination SEL-212 product received sequential IV administrations of ImmTOR immediately followed by pegadricase, with the exception of the cohort of patients that received 0.4 mg/kg pegadricase followed 48 h later by an IV infusion of 0.03 mg/kg ImmTOR. Patients were pre-medicated with oral 60 mg fexofenadine to reduce the potential for infusion reactions. Sera were collected at baseline, 0.25 h, 0.5 h, 1 h, 1.5 h, 2 h, 2.5 h, 3 h, 6 h, 9 h, 12 h, 24 h, day 3, day 7, day 14, day 21, and day 30 for analysis of sUA and at baseline, day 14, day 21, and day 30 for analysis of ADAs. Patients that maintained sUA < 6 mg/dL at day 30 were invited to return on a voluntary basis for follow-up visits to monitor sUA and ADA levels after day 30.

### Anti-drug antibody assays

A validated cGLP sandwich ELISA method was used for the determination of anti-uricase IgG antibodies in human serum. Analysis was conducted at a central lab that was blinded to the treatment. Uricase (non-pegylated, 3SBio) coated on to an ELISA plate was used to capture anti-uricase antibodies in human serum using a minimum required dilution (MRD) of 1:40. Captured anti-uricase antibodies were colorimetrically detected using a goat anti-human IgG Fc antibody conjugated to HRP (Abcam, ab98624) and TMB substrate. The optical density (O.D.) at 450 nm minus the O.D. at 570 nm was reported. Samples with reported O.D. values above the floating cut point (negative control O.D. x 2.147) were defined as positive. The mass-based sensitivity of the assay was assessed using a custom chimeric antibody (variable region from a murine mAb raised against uricase and constant domain from human IgG1) produced at Genscript. Five replicate titration curves were used to estimate sensitivity by spiking the positive control antibody (PC) into neat serum at 40 ng/mL and diluted 1:40. The spiked samples were then serially diluted 1:3 in sample buffer containing 2.5% serum to create 8 dilutions. The antibody concentrations from the serially diluted curves that crossed the cut point was then interpolated. The mean of the antibody concentrations was obtained and multiplied by the MRD of 1:40 to obtain a sensitivity of 2 ng/mL in neat serum (Supplementary Fig. 3). To determine drug tolerance, the PC was spiked into neat serum such that the ability to detect 200, 100 and 50 ng/mL of PC in the presence of a range of uricase concentrations (0.07–50 ug/mL) was assessed. The 200, 100 and 50 ng/mL of PC spikes signaled above the cut point in the presence of 50 ug/mL of PC, indicating drug tolerance well above the C_max_ value reported for the highest dose of pegadricase administered in the clinical study (Supplementary Fig. 4).

A validated cGLP sandwich ELISA method was used for the determination of anti-PEG antibodies. Analysis was conducted at a central lab that was blinded to the treatment. PEG-30k (Nanocs) coated on to an ELISA plate was used to capture anti-PEG antibodies in human serum. Captured anti-PEG antibodies were colorimetrically detected using a rabbit anti-human IgG and IgM antibody conjugated to HRP (Jackson ImmunoResearch, #309-035-107) and TMB substrate. The optical density (O.D.) at 450 nm minus the O.D. at 570 nm was reported. Samples with reported O.D. values above a fixed cut point of 0.213 were defined as positive.

### Serum uricase activity

A validated EIA analytical method was used for the determination of pegadricase in human serum. The analytical assay was conducted in accordance with the relevant parts of ICH guideline for Good Clinical Practice (GCP) (CPMP/ICH/135/95) and performed at a central lab that was blinded to the treatment. Serum uricase activity was assessed using an enzyme immunoassay (EIA) with fluorimetry detection. The assay was performed in a 96-well microtiter plate. Uric acid reacts with water and oxygen in the presence of the enzyme uricase to produce allantoin and H_2_O_2_. In the presence of horseradish peroxidase, a fluorescence probe reacts with H_2_O_2_ in a 1:1 stoichiometry to produce a fluorescent product that was read by a fluorescence microplate reader with an excitation of 530 nm and an emission of 590 nm. Fluorescence values are proportional to the uricase concentration or concentration in unknown samples determined by comparison with its respective standard curve.

### Reporting summary

Further information on research design is available in the Nature Research Reporting Summary linked to this article.

## Supplementary information

Supplementary Information

Reporting Summary

## Data Availability

Primary data, including de-identified individual patient data, are provided in the accompanying Source Data file. Additional information about the conduct of the trial will be provided upon reasonable request. Source data are provided with this paper.

## Source data

Source Data
