# Supplementary material for: Tolerogenic nanoparticles mitigate the formation of anti-drug antibodies against pegylated uricase in patients with hyperuricemia
Source: Nat Commun. 2022 Jan 12;13:272. doi: 10.1038/s41467-021-27945-7 (PMC8755849; doi:10.1038/s41467-021-27945-7)
Supplement: Supplementary file 1 — Supplementary Information [file 41467_2021_27945_MOESM1_ESM.pdf]

Tolerogenic Nanoparticles Mitigate the Formation of Anti-Drug Antibodies against Pegylated Uricase in Patients with Hyperuricemia

First author: Earl Sands

Corresponding author: Takashi Kei Kishimoto (kkishimoto@selectabio.com)

Supplemental Figure 1

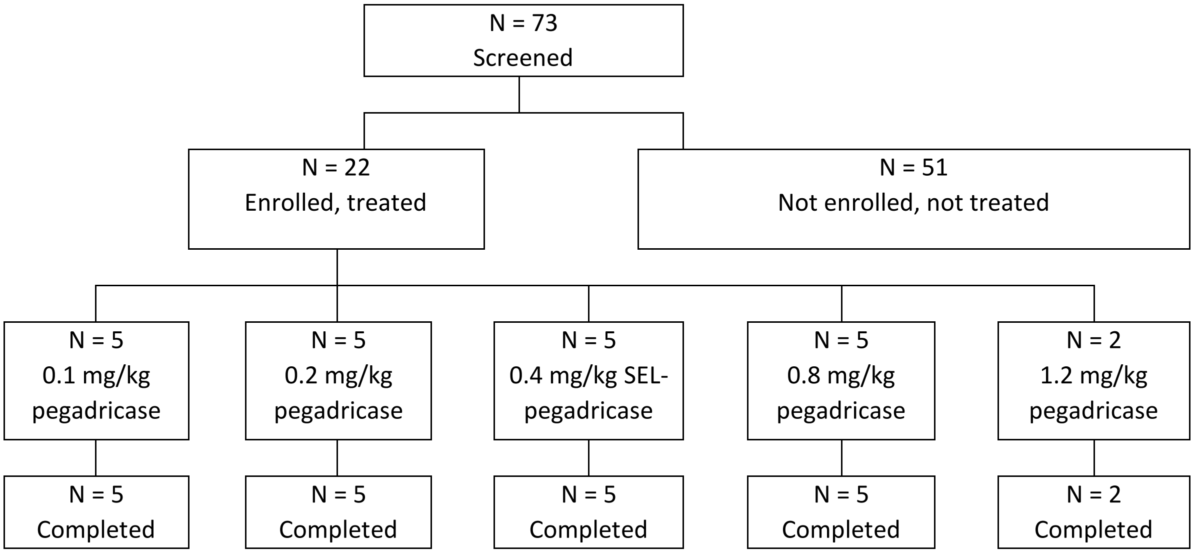

Supplementary Figure 1. Phase 1a patient flow diagram. SEL-037/101 Phase 1a Trial (NCT02464605) was a multi-center, open label, sequential, single-ascending-dose study to assess the safety, tolerability, activity, and immunogenicity of pegadricase. Seventy-three male and female patients, 21-70 years of age, were screened for sUA levels >6mg/dL. Twenty-five patients were planned to be enrolled, and 22 patients were actually enrolled in the study. After clinical review of sUA data from the 0.2, 0.4, and 0.8 mg/kg cohorts revealed that there was no further extension in the duration of uric acid lowering beyond the 0.2 mg/kg pegadricase dose, thus a decision was made to prematurely stop enrollment of subjects in the 1.2 mg/kg group. At the time the decision was made, 2 subjects had already been dosed with 1.2 mg/kg pegadricase. All enrolled subjects completed the study.

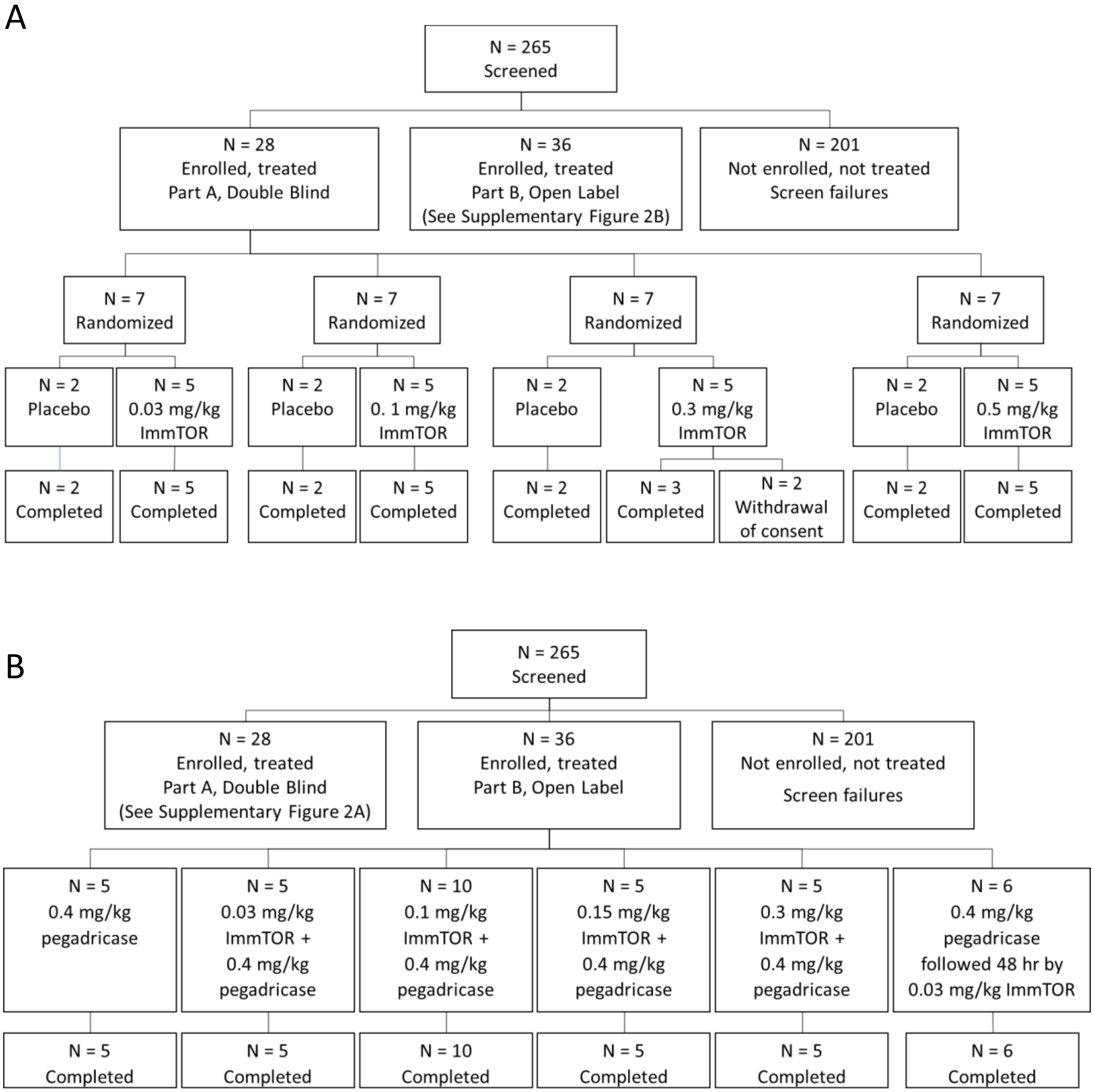

Supplementary Figure 2. Phase 1b patient flow diagram. SEL-212/101 was a multi-center, double-blind, single-ascending-dose study of ImmTOR integrated with an open label, single-ascending-dose study of SEL 212 (ascending doses of ImmTOR combined with a fixed 0.4 mg/kg dose of pegadricase) to assess the safety, tolerability, activity, and immunogenicity of SEL 212. Sixty-four patients were enrolled, and 62 completed the study. A) In the first part of the study, four cohorts of 7 patients received treatment with placebo or escalating doses of ImmTOR (0.03 mg/kg, 0.1 mg/kg, 0.3 mg/kg, 0.5 mg/kg). The 7 subjects in each cohort were blinded and randomized to ImmTOR (5 patients) or placebo (2 patients). Investigators were blinded, but the site pharmacist and the nurse administering the IV syringe infusion were not blinded. B) In the second part of the study, which was open label, patients were assigned to receive a single IV infusion of pegadricase alone (0.4 mg/kg) alone, or SEL-212 (0.03, 0.1, 0.15, or 0.3 mg/kg ImmTOR combined with 0.4 mg/kg pegadricase.

Supplementary Figure 3.

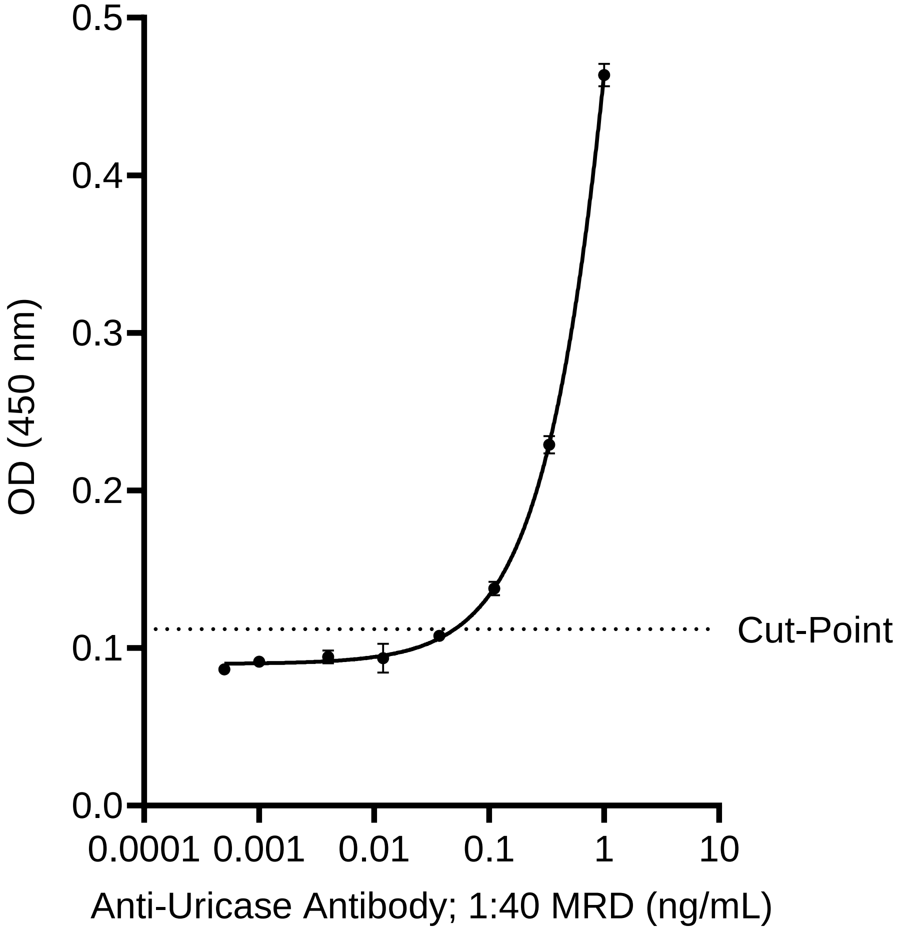

Supplementary Figure 3. Determination of Anti-Uricase Antibody Assay Sensitivity. a) The positive control anti-uricase antibody was spiked into neat serum and diluted 1:40, the minimum required dilution (MRD), and analyzed in the sandwich ELISA to create 5 replicate curves. The concentrations at which the curves crossed the cut-point were interpolated and the mean interpolated value was obtained. The sensitivity of the assay was then calculated by multiplying the mean interpolated value by 40 (the MRD). b) The average OD (450 nm) values obtained for each anti-uricase antibody concentration analyzed, was graphed showing the point at which the cut-point was crossed. Source data are provided as a Source Data file.

Supplementary Figure 4.

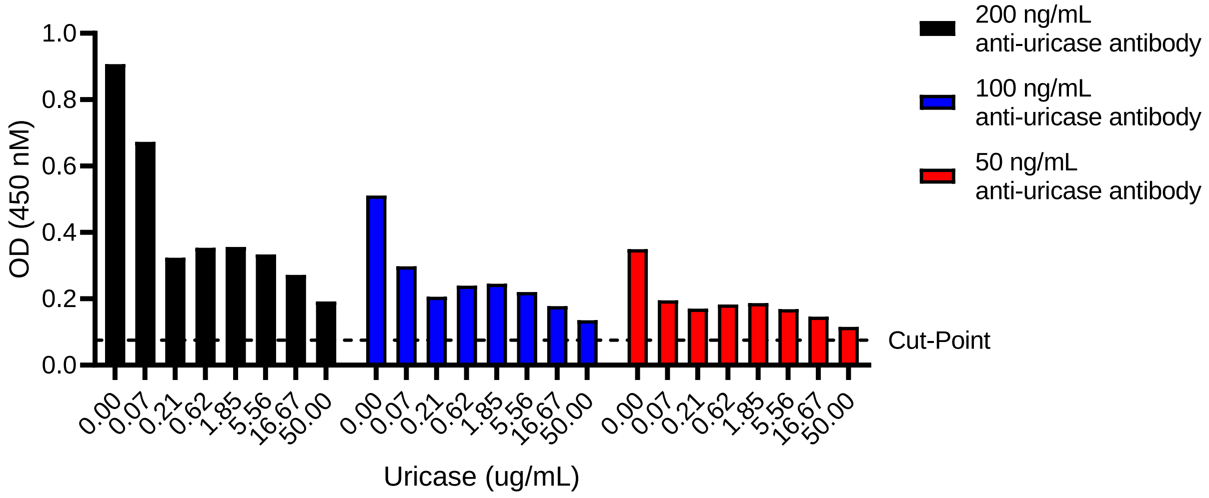

Supplementary Figure 4. Determination of Drug Tolerance of the Anti-Uricase Antibody Assay. a) Three different concentrations of anti-uricase antibody (200, 100 and 50 ng/mL) were incubated with increasing amounts of uricase in neat serum to determine what concentration would inhibit the unspiked OD value of the sandwich ELISA, below the cut-point. The highest amount of uricase spiked (50 ug/mL) did not inhibit any of the anti-uricase antibody concentrations tested below the cut-point. This demonstrated a drug tolerance level of at least 50 ug/mL of uricase at a concentration of 50 ng/mL of anti-uricase antibody. This is above the estimated Cmax of 18 µg/mL at the highest dose of SEL-037. b) The inhibition of the anti-uricase antibody to bind to the uricase coated on the well by the increasing concentration of free uricase is shown in the graph via the decrease in OD (450 nm). Significant inhibition was observed, however, at the maximum concentration of uricase tested (50 µg/mL), the signal did not drop below the cut-point. Source data are provided as a Source Data file.

Supplemental Table 1. Phase 1a and 1b patient inclusion and exclusion criteria

Type	Criteria
Inclusion	Evidence of a personally signed and dated informed consent document indicating that subject has been informed of all pertinent aspects of the study
Inclusion	Male or female subjects ages 21 to 75 inclusive (Phase 1a) or 21-70 (Phase 1b)
Inclusion	Female subjects must be of non-childbearing potential or agree to use of double contraceptive
Inclusion	Has at the Screening Visit a serum uric acid ≥ 6 mg/dL, with or without a history of gout
Inclusion	The use of allopurinol, febuxostat (Uloric®), or probenecid as uric acid-lowering therapy is permissible if dosing has been stable for at least the month prior to the Screening Visit
Inclusion	Negative for anti-PEG antibodies at Screening Visit
Inclusion	Human immunodeficiency virus (HIV) and hepatitis C negative
Inclusion	Has adequate venous access and able to receive IV therapy
Exclusion	Prior exposure to any experimental or marketed uricase
Exclusion	History of anaphylaxis or severe allergy, or any allergy to pegylated products
Exclusion	History of hematological or autoimmune disorders, is immunosuppressed or immunocompromised
Exclusion	Unstable symptomatic gout that would likely require a change in uric acid-lowering medication
Exclusion	Initiation or change in dose of hormone-replacement therapy for menopausal women 1 month prior to Screening or during Screening Phase
Exclusion	Abnormal, clinically-significant clinical laboratory values, except serum uric acid
Exclusion	History of coronary artery disease, myocardial infarction, congestive heart disease or ongoing treatment for arrhythmia
Exclusion	Use of non-selective beta-adrenergic antagonist or anticoagulants
Exclusion	Uncontrolled diabetes (baseline HbA1c ≥8%) or blood pressure (>140/90 (phase 1a); >160/95 Phase 1b)
Exclusion	Fasting triglyceride level > 200 mg/dL; fasting LDL cholesterol > 160 mg/dL (Phase 1b only)
Exclusion	Use of cytochrome P450 3A4 (CYP3A4) and/or p-glycoprotein (PGP) inhibitors or inducers (Phase 1b only)
Exclusion	Use of drugs known to interact with Rapamune® (Phase 1b only)

Supplemental Table 2. Phase 1a Patient Demographics

	**0.1 mg/kg Pegadricase**	**0.2 mg/kg Pegadricase**	**0.4 mg/kg Pegadricase**	**0.8 mg/kg Pegadricase**	**1.2 mg/kg Pegadricase**	**All Subjects**
Subjects entered<br>n<br>(male/female)	5<br>(5/0)	5<br>(4/1)	5<br>(3/2)	5<br>(5/0)	2<br>(1/1)	22<br>(18/4)
Study completion<br>n<br>(%)	5<br>(100)	5<br>(100)	5<br>(100)	5<br>(100)	2<br>(100)	22<br>(100)
Median age, years<br>(range)	54.0<br>(41; 67)	51.0<br>(41; 63)	54.0<br>(37; 57)	51.0<br>(29; 59)	52.5<br>(45; 60)	51.5<br>(29; 67)
Median body mass index<br>kg/m^2<br>(range)	36.3<br>(24.3; 37.3)	30.1<br>(26.6; 34.2)	32.0<br>(27.0; 33.9)	30.0<br>(28.7; 33.7)	31.8<br>(30.2; 33.3)	30.2<br>(24.3; 37.3)
Race, n (%)<br>White<br>Black or African American	 5 (100)<br>0	 4 (80.0)<br>1 (20.0)	 4 (80.0)<br>1 (20.0)	 3 (60.0)<br>2 (40.0)	 2 (100)<br>0 (0.0)	 18 (81.8)<br>4 (18.2)

mg – milligram; kg – kilograms, , m – meter, n - number

Supplementary Table 3. Phase 1a Treatment Emergent Adverse Events (TEAEs)

**Treatment-Emergent Adverse Events**	**0.1 mg/kg Pegadricase N = 5**	**0.2 mg/kg Pegadricase N = 5**	**0.4 mg/kg Pegadricase N = 5**	**0.8 mg/kg Pegadricase N = 5**	**1.2 mg/kg Pegadricase N = 2**	**All Subjects N = 22**
Most frequently reported TEAEs						
Gout, n (%)	3 (60.0)	1 (20.0)	0	0	0	4 (18.2)
Arthralgia, n (%)	0	0	1 (20.0)	0	1 (50.0)	2 (9.1)
Total TEAEs, n (%)	3 (60.0)	2 (40.0)	2 (40.0)	1 (20.0)	2 (100)	10 (45.5)
Deaths, n (%)	0	0	0	0	0	0
Serious TEAEs, n (%)	0	0	0	0	0	0
Treatment-related TEAE, n (%)	2 (40.0)	2 (40.0)	2 (40.0)	0	1 (50.0)	7 (31.8)
TEAE leading to treatment discontinuation, n (%)	0	0	0	0	0	0

TEAE – Treatment emergent adverse event; mg – milligram; kg – kilograms, N = number of subjects with data; n = number of subjects with that observation

Supplemental Table 4. Phase 1b Patient Demographics

		**Placebo** N=8	**0.03 mg/kg ImmTOR** N=5	**0.1 mg/kg ImmTOR** N=5	**0.3 mg/kg ImmTOR** N=5	**0.5 mg/kg ImmTOR** N=5	**0.4 mg/kg pegadricase** N=5	**0.03 mg/kg ImmTOR + pegadricase** N=5	**0.1 mg/kg ImmTOR + pegadricase** N=10	**0.15 mg/kg ImmTOR + pegadricase** N=5	**0.3 mg/kg ImmTOR + pegadricase** N=5	**Pegadricase + 0.03 mg/kg ImmTOR** N=6
**Age**, years	Mean (SD)	50.6 (8.1)	50.0 (10.4)	50.2 (11.8)	53.0 (12.6)	45.8 (14.8)	45.8 (14.8)	45.0 (15.8)	47.9 (9.0)	49.8 (17.0)	46.0 (6.9)	56.7 (7.6)
	Min, Max	39, 62	41, 67	38, 67	34, 65	26, 62	26, 62	31, 69	34, 59	29, 70	36, 54	48, 65
**Gender**												
Male	n (%)	8 (100)	5 (100)	5 (100)	5 (100)	4 (80.0)	5 (100)	5 (100)	9 (90.0)	5 (100)	5 (100)	6 (100)
Female	n (%)	0	0	0	0	1 (20.0)	0	0	1 (10.0)	0	0	0
**Race**												
Asian	n (%)	1 (12.5)	0	0	0	0	0	0	0	2 (40.0)	0	0
Black or African American	n (%)	2 (25.0)	1 (20.0)	3 (60.0)	0	0	0	4 (80.0)	7 (70.0)	1 (20.0)	2 (40.0)	0
White	n (%)	5 (62.5)	4 (80.0)	2 (40.0)	5 (100)	5 (100)	5 (100)	1 (20.0)	3 (30.0)	2 (40.0)	3 (60.0)	5 (83.3)
Other	n (%)	0	0	0	0	0	0	0	0	0	0	1 (16.7)
**Ethnicity**												
Hispanic or Latino	n (%)	3 (37.5)	0	2 (40.0)	2 (40.0)	2 (40.0)	2 (40.0)	2 (40.0)	1 (10.0)	2 (40.0)	2 (40.0)	5 (83.3)
Not Hispanic or Latino	n (%)	5 (62.5)	5 (100)	3 (60.0)	3 (60.0)	3 (60.0)	3 (60.0)	3 (60.0)	9 (90.0)	3 (60.0)	3 (60.0)	1 (16.7)
**Height**, cm	Mean (SD)	174.3 (4.4)	178.8 (4.9)	177.5 (6.9)	174.2 (10.7)	174.6 (7.7)	177.6 (9.5)	174.0 (7.6)	175.2 (9.4)	172.1 (12.4)	181.6 (12.2)	169.3 (5.5)
	Min, Max	165.0, 177.8	173.0, 185.0	170.0, 185.0	162.7, 188.9	161.6, 181.0	165.0, 188.0	166.0, 184.0	164.5, 192.0	157.5, 183.0	170.0, 202.0	162.2, 177.3
**Weight**, kg	Mean (SD)	98.2 (11.5)	96.9 (16.9)	89.2 (17.7)	86.3 (6.3)	94.6 (2.8)	88.5 (5.0)	96.6 (22.9)	97.1 (10.7)	97.1 (31.4)	115.3 (32.1)	96.5 (20.4)
	Min, Max	81.6, 120.1	76.0, 122.2	72.2, 116.9	77.2, 94.7	90.2, 96.6	84.3, 94.2	73.3, 128.9	79.3, 107.2	61.0, 135.8	77.8, 158.1	66.7, 122.1
**Body Mass Index**, kg/m²	Mean (SD)	32.4 (4.6)	30.2 (4.3)	28.6 (7.1)	28.8 (4.6)	31.2 (3.5)	28.18 (2.0)	32.1 (8.4)	31.9 (5.0)	32.08 (6.4)	34.5 (6.2)	33.6 (6.8)
	Min, Max	25.8, 39.1	25.4, 37.1	21.1, 40.4	23.8, 35.8	27.8, 37.0	26.7, 31.4	21.7, 44.1	24.5, 38.5	24.6, 40.6	25.3, 40.6	24.5, 41.7

mg – milligram; kg – kilograms; N = number of subjects with data; n = number of subjects with that observation; SD – standard deviation, Min – minimum; Max – maximum

Supplemental Table 5. Phase 1b Anti-uricase IgG and anti-PEG antibodies

		Anti-uricase IgG				Anti-peg		
Cohort	Patient	Predose	Day 14	Day 21	Day 30	Day 14	Day 21	Day 30
**Cohort A** 0.4 mg/kg pegadricase	103-0015	BDL	29,160	29,160	9,720	38,462	8,741	3,013
	104-0032	BDL	3,240	1,080	1,080	BDL	BDL	BDL
	104-0036	BDL	29,160	9,720	9,720	BDL	BDL	BDL
	108-0010	BDL	1,080	1,080	1,080	30,378	7,408	3301
	109-0012	BDL	3,240	3,240	1,080	993	874	BDL
**Cohort F** 0.03 mg/kg ImmTOR + 0.4 mg/kg pegadricase	104-0012	BDL	40	40	120	BDL	BDL	BDL
	104-0016	BDL	360	1,080	3,240	BDL	BDL	BDL
	104-0017	BDL	3,240	3,240	1,080	BDL	BDL	BDL
	107-0016	BDL	120	360	1,080	BDL	BDL	BDL
	108-0001	BDL	29,160	29,160	29,160	BDL	BDL	BDL
**Cohort G** 0.1 mg/kg ImmTOR + 0.4 mg/kg pegadricase	102-0005	BDL	40	BDL	BDL	BDL	BDL	BDL
	104-0027	BDL	1,080	29,160	29,160	BDL	BDL	BDL
	107-0018	BDL	BDL	BDL	BDL	BDL	BDL	BDL
	107-0021	BDL	BDL	BDL	BDL	BDL	BDL	BDL
	108-0008	BDL	BDL	BDL	120	BDL	BDL	BDL
	106-0004	BDL	BDL	BDL	BDL	BDL	BDL	BDL
	111-0018	BDL	BDL	40	120	BDL	336	447
	111-0022	BDL	120	360	360	338	BDL	419
	111-0028	BDL	BDL	BDL	BDL	BDL	BDL	BDL
	111-0029	BDL	9,720	87,480	9,720	BDL	1515	1445
**Cohort H** 0.15 mg/kg ImmTOR + 0.4 mg/kg pegadricase	104-0091	BDL	BDL	BDL	BDL	BDL	BDL	BDL
	104-0094	BDL	BDL	BDL	BDL	BDL	BDL	BDL
	111-0043	BDL	BDL	BDL	BDL	BDL	BDL	BDL
	111-0045	BDL	BDL	BDL	BDL	BDL	BDL	BDL
	111-0049	120	40	120	9,720	BDL	BDL	BDL
**Cohort I** 0.3 mg/kg ImmTOR + 0.4 mg/kg pegadricase	103-0019	BDL	BDL	BDL	BDL	BDL	BDL	BDL
	104-0050	BDL	BDL	BDL	BDL	BDL	BDL	BDL
	104-0060	120	120	120	120	BDL	BDL	BDL
	107-0027	BDL	BDL	BDL	BDL	BDL	BDL	BDL
	107-0028	BDL	BDL	BDL	BDL	BDL	BDL	BDL
**Cohort J** 0.4 mg/kg pegadricase + 0.03 mg/kg ImmTOR	104-0080	BDL	BDL	120	120	BDL	BDL	BDL
	104-0081	BDL	BDL	BDL	40	BDL	BDL	BDL
	104-0086	BDL	87,480	87,480	29,160	474	759	607
	104-0090	BDL	360		1,080	BDL		BDL
	106-0001	BDL	120	1,080	9,720	BDL	BDL	BDL
	106-0003	BDL	360	3,240	87,480	442	2295	1316

BDL – below detectables

Supplemental Table 6. Phase 1b Detailed TEAEs

	Placebo	ImmTOR				Pegadricase	SEL-212				
**ImmTOR (mg/kg)**	**0**	**0.03**	**0.1**	**0.3**	**0.5**	**0**	**0.03**	**0.1**	**0.15**	**0.3**	**0.03***
**Pegadricase (mg/kg)**	**0**	**0**	**0**	**0**	**0**	**0.4**	**0.4**	**0.4**	**0.4**	**0.4**	**0.4***
N	8	5	5	5	5	5	5	10	5	5	6
**Subjects with at least 1 TEAE**	**2 (25.0)**	**3 (60.0)**	**4 (80.0)**	**4 (80.0)**	**5 (100)**	**3 (60.0)**	**2 (40.0)**	**9 (90.0)**	**3 (60.0)**	**5 (100)**	**2 (33.3)**
**Nervous System Disorders, n (%)**	**1 (12.5)**	**1 (20.0)**	**2 (40.0)**	**3 (60.0)**	**5 (100)**	**0**	**0**	**0**	**1 (20.0)**	**3 (60.0)**	**2 (33.3)**
Headache	1 (12.5)	1 (20.0)	1 (20.0)	3 (60.0)	2 (40.0)	0	0	0	0	3 (60.0)	1 (16.7)
Dizziness	1 (12.5)	0	0	0	2 (40.0)	0	0	0	1 (20.0)	1 (20.0)	1 (16.7)
Paraesthesia	0	0	1 (20.0)	0	1 (20.0)	0	0	0	0	0	0
Hypoaesthesia	0	0	0	0	1 (20.0)	0	0	0	0	0	0
Dysaesthesia	0	0	0	0	0	0	0	0	0	0	1 (16.7)
Restless legs syndrome	0	0	0	0	0	0	0	0	0	1 (20.0)	0
Migraine	0	0	0	0	1 (20.0)	0	0	0	0	0	0
**Skin and subcutaneous tissue disorders, n (%)**	**0**	**0**	**1 (20.0)**	**1 (20.0)**	**3 (60.0)**	**1 (20.0)**	**0**	**3 (30.0)**	**2 (40.0)**	**4 (80.0)**	**2 (33.3)**
Pruritus	0	0	0	1 (20.0)	0	0	0	0	0	0	1 (16.7)
Pruritis allergic	0	0	0	0	0	0	0	0	0	1 (20.0)	0
Rash	0	0	0	0	0	0	0	1 (10.0)	1 (20.0)	1 (20.0)	1 (16.7)
Rash generalized	0	0	0	0	0	0	0	1 (10.0)	0	0	0
Rash maculo-papular	0	0	0	0	2 (40.0)	0	0	0	0	0	0
Rash pruritic	0	0	0	0	0	0	0	1 (10.0)	1 (20.0)	0	0
Acne	0	0	0	0	1 (20.0)	0	0	0	0	0	0
Erythema	0	0	1 (20.0)	0	0	0	0	0	0	0	0
Dermatitis	0	0	0	0	0	1 (20.0)	0	0	0	0	0
Hyperhidrosis	0	0	0	0	0	0	0	0	0	1 (20.0)	0
Urticaria	0	0	0	0	0	0	0	0	0	1 (20.0)	0
**Gastrointestinal disorders, n (%)**	**0**	**0**	**2 (40.0)**	**0**	**3 (60.0)**	**0**	**0**	**3 (30.0)**	**2 (40.0)**	**3 (60.0)**	**0**
Nausea	0	0	1 (20.0)	0	0	0	0	0	1 (20.0)	2 (40.0)	0
Aphthous ulcer	0	0	0	0	1 (20.0)	0	0	1 (10.0)	0	1 (20.0)	0
Stomatitis	0	0	0	0	2 (40.0)	0	0	1 (10.0)	0	0	0
Diarrhea	0	0	1 (20.0)	0	0	0	0	0	0	1 (20.0)	0
Toothache	0	0	1 (20.0)	0	0	0	0	0	0	0	0

Vomiting	0	0	0	0	1 (20.0)	0	0	0	0	0	0
Abdominal discomfort	0	0	0	0	0	0	0	1 (10.0)	0	0	0
Abdominal pain	0	0	0	0	0	0	0	0	0	1 (20.0)	0
Constipation	0	0	0	0	0	0	0	0	0	1 (20.0)	0
Intestinal mass	0	0	0	0	0	0	0	0	1 (20.0)	0	0
Oral mucosa erythema	0	0	0	0	0	0	0	0	0	1 (20.0)	0
Small intestinal obstruction	0	0	0	0	0	0	0	0	1 (20.0)	0	0
Upper gastrointestinal haemorrhage	0	0	0	0	0	0	0	0	1 (20.0)	0	0
**Infections and Infestations, n (%)**	**0**	**0**	**2 (40.0)**	**1 (20.0)**	**1 (20.0)**	**0**	**1 (20.0)**	**4 (40.0)**	**0**	**0**	**0**
Pneumonia	0	0	0	0	1 (20.0)	0	0	2 (20.0)	0	0	0
Conjunctivitis	0	0	1 (20.0)	0	0	0	0	0	0	0	0
Tonsilitis	0	0	0	1 (20.0)	0	0	0	0	0	0	0
Upper respiratory tract infection	0	0	0	0	1 (20.0)	0	0	0	0	0	0
Viral upper respiratory tract infection	0	0	1 (20.0)	0	0	0	0	0	0	0	0
Vulvovaginal candidiasis	0	0	0	0	1 (20.0)	0	0	0	0	0	0
Bronchitis viral	0	0	0	0	0	0	0	1 (10.0)	0	0	0
Nasopharyngitis	0	0	0	0	0	0	1 (20.0)	0	0	0	0
Pharyngitis	0	0	0	0	0	0	0	1 (10.0)	0	0	0
Sinusitis	0	0	0	0	0	0	0	1 (10.0)	0	0	0
**Injury, poisoning and procedural complications, n (%)**	**0**	**1 (20.0)**	**1 (20.0)**	**1 (20.0)**	**2 (40.0)**	**0**	**0**	**0**	**0**	**0**	**0**
Infusion related reaction	0	1 (20.0)	1 (20.0)	1 (20.0)	1 (20.0)	0	0	0	0	0	0
Hair injury	0	0	0	0	1 (20.0)	0	0	0	0	0	0

TEAE – Treatment emergent adverse event; mg – milligram; kg – kilograms, N = number of subjects with data; n = number of subjects with that observation

*Patients in this cohort were administered 0.4 mg/kg pegadricase followed 48 hr later by 0.03 mg/kg ImmTOR

Supplemental Table 7. Phase 1b TEAE severity and relationship to study drug

	**Placebo**	**ImmTOR**				**Pegadricase**	**SEL-212**				
**ImmTOR (mg/kg)**	**0**	**0.03**	**0.1**	**0.3**	**0.5**	**0**	**0.03**	**0.1**	**0.15**	**0.3**	**0.03***
**Pegadricase (mg/kg)**	**0**	**0**	**0**	**0**	**0**	**0.4**	**0.4**	**0.4**	**0.4**	**0.4**	**0.4***
N	8	5	5	5	5	5	5	10	5	5	6
**Subjects with at least 1 TEAE**	**2 (25.0)**	**3 (60.0)**	**4 (80.0)**	**4 (80.0)**	**5 (100)**	**3 (60.0)**	**2 (40.0)**	**9 (90.0)**	**3 (60.0)**	**5 (100)**	**2 (33.3)**
Mild	2 (25.0)	2 (40.0)	4 (80.0)	2 (40.0)	1 (20.0)	2 (40.0)	1 (20.0)	5 (50.0)	2 (40.0)	3 (60.0)	1 (16.7)
Moderate	0	1 (20.0)	0	2 (40.0)	2 (40.0)	1 (20.0)	1 (20.0)	4 (40.0)	0	2 (40.0)	1 (16.7)
Severe	0	0	0	0	2 (40.0)	0	0	0	1 (20.0)	0	0
Life-threatening	0	0	0	0	0	0	0	0	0	0	0
Not related to study drug	0	0	1 (20.0)	0	0	2 (40.0)	0	3 (30.0)	0	1 (20.0)	1 (16.7)
Unlikely to be related	1 (12.5)	1 (20.0)	1 (20.0)	1 (20.0)	0	0	1 (20.0)	1 (10.0)	1 (20.0)	1 (20.0)	0
Possibly related	1 (12.5)	1 (20.0)	0	2 (40.0)	4 (80.0)	1 (20.0)	0	5 (50.0)	2 (40.0)	2 (40.0)	1 (16.7)
Related	0	1 (20.0)	2 (40.0)	1 (20.0)	1 (20.0)	0	1 (20.0)	0	0	1 (20.0)	0

*Patients in this cohort were administered 0.4 mg/kg pegadricase followed 48 hr later by 0.03 mg/kg ImmTOR
