# Supplementary material for: Tolerogenic nanoparticles mitigate the formation of anti-drug antibodies against pegylated uricase in patients with hyperuricemia
Source: Nat Commun. 2022 Jan 12;13:272. doi: 10.1038/s41467-021-27945-7 (PMC8755849; doi:10.1038/s41467-021-27945-7)
Supplement: Supplementary file 2 — Reporting Summary [file 41467_2021_27945_MOESM2_ESM.pdf]

nature research

Corresponding author(s): Takashi Kei Kishimoto

Last updated by author(s): 11 Dec 2021

Reporting Summary

Nature Research wishes to improve the reproducibility of the work that we publish. This form provides structure for consistency and transparency in reporting. For further information on Nature Research policies, see Authors & Referees and the Editorial Policy Checklist .

Statistics

For all statistical analyses, confirm that the following items are present in the figure legend, table legend, main text, or Methods section.

n/a	Confirmed	
☐	☒	The exact sample size (*n*) for each experimental group/condition, given as a discrete number and unit of measurement
☐	☒	A statement on whether measurements were taken from distinct samples or whether the same sample was measured repeatedly
☒	☐	The statistical test(s) used AND whether they are one- or two-sided<br>*Only common tests should be described solely by name; describe more complex techniques in the Methods section.*
☒	☐	A description of all covariates tested
☐	☒	A description of any assumptions or corrections, such as tests of normality and adjustment for multiple comparisons
☒	☐	A full description of the statistical parameters including central tendency (e.g. means) or other basic estimates (e.g. regression coefficient) AND variation (e.g. standard deviation) or associated estimates of uncertainty (e.g. confidence intervals)
☒	☐	For null hypothesis testing, the test statistic (e.g. *F*, *t*, *r*) with confidence intervals, effect sizes, degrees of freedom and *P* value noted<br>*Give P values as exact values whenever suitable.*
☒	☐	For Bayesian analysis, information on the choice of priors and Markov chain Monte Carlo settings
☒	☐	For hierarchical and complex designs, identification of the appropriate level for tests and full reporting of outcomes
☒	☐	Estimates of effect sizes (e.g. Cohen's *d*, Pearson's *r*), indicating how they were calculated

Our web collection on statistics for biologists contains articles on many of the points above.

Software and code

Policy information about availability of computer code

Data collection	Bioanalytical data were collected on a Molecular Devices plate reader using SoftMax Pro software vGxP 5.3
Data analysis	SAS version 9.2 (SAS Institute Cary, NC) was used for programming; Prism v8.2 (Graphpad, San Diego, CA)

For manuscripts utilizing custom algorithms or software that are central to the research but not yet described in published literature, software must be made available to editors/reviewers. We strongly encourage code deposition in a community repository (e.g. GitHub). See the Nature Research guidelines for submitting code & software for further information.

Data

Policy information about availability of data

All manuscripts must include a data availability statement. This statement should provide the following information, where applicable:
- Accession codes, unique identifiers, or web links for publicly available datasets
- A list of figures that have associated raw data
- A description of any restrictions on data availability

De-identified individual patient data for all figures are now included in the accompanying Source Data file

Field-specific reporting

Please select the one below that is the best fit for your research. If you are not sure, read the appropriate sections before making your selection.

☒ Life sciences ☐ Behavioural & social sciences ☐ Ecological, evolutionary & environmental sciences

For a reference copy of the document with all sections, see nature.com/documents/nr-reporting-summary-flat.pdf

Life sciences study design

All studies must disclose on these points even when the disclosure is negative.

Sample size	Pegylated uricases are published to induce anti-drug antibodies (ADAs) in over 90% of patients (Sundy et al, JAMA, 2011). In the current Phase 1a study of pegadricase alone, 20 of 22 patients (90.9%) developed ADA titers greater than 1:100 and had serum uric acid levels (sUA) return to baseline line levels by day 30. The remaining two patients showed titers of 1:40 and maintained low sUA levels for 30 days. Thus we determined that cohorts of 5 patients in the Phase 1b evaluating pegadricase combined with ImmTOR would be sufficient to demonstrate inhibition of anti-drug antibodies and show corresponding sustained reduction of sUA for 30 days, based on the degree of efficacy observed in pre-clinical studies (Kishimoto et al, Nature Nanotech, 2016).
Data exclusions	No data were excluded
Replication	The Phase 1a study showed that 4 of 5 subjects dosed with 0.4 mg/kg pegadricase developed ADA titers greater than 1:1000 correlating with sUA returning to baseline by 30 days (Figure 2B). This observation was replicated in an independent cohort of subjects in the Phase 1 b study with 4 of 5 subjects dosed with 0.4 mg/kg pegadricase alone developing high titer ADAs which correlated with sUA levels returning to baseline by 30 days (Figure 4, Cohort E). In the unpublished Phase 2 clinical trial, we similarly had a group of 6 patients that were treated with pegadricase alone. 5 of the 6 subjects developed high titer ADAs greater correlating with sUA levels returning to baseline by 30 days. All attempts to replicate the data were successful (i.e. there was no other attempt to replicate the data).
Randomization	The Phase 1a study was a open-label, sequential, single ascending dose study of pegadricase. Patients were assigned in the order of enrollment to the next available cohort in this open label ascending dose trial. In the Phase 1b study, the safety of ImmTOR was assessed in a double-blind placebo controlled manner in which cohorts of 7 patients were randomized to treatment with ImmTOR (5 patients per cohort) or to placebo (2 patients per cohort). However, the assessment of cohorts dosed with SEL-212 (combination of ImmTOR + pegadricase) was performed in an open-label, sequential cohort manner.
Blinding	Investigators were only blinded to the dosing of ImmTOR versus placebo in the Phase 1b study, but not to the dosing of SEL-212, which was open-label. Blinding to ImmTOR was conducted to assess safety of ImmTOR. Dosing of SEL-212 was not blinded as the clinical investigators were not involved in the assessment of the bioanalytical endpoints of sUA levels, serum uricase activity, and anti-uricase antibodies. The bioanalytical assessments of serum uric acid levels, serum uricase activity, and anti-uricase antibodies were performed at Contract research Organizations that were blinded to treatment groups.

Reporting for specific materials, systems and methods

We require information from authors about some types of materials, experimental systems and methods used in many studies. Here, indicate whether each material, system or method listed is relevant to your study. If you are not sure if a list item applies to your research, read the appropriate section before selecting a response.

Materials & experimental systems

n/a	Involved in the study	
☐	☒	Antibodies
☒	☐	Eukaryotic cell lines
☒	☐	Palaeontology
☒	☐	Animals and other organisms
☐	☒	Human research participants
☐	☒	Clinical data

Methods

n/a	Involved in the study	
☒	☐	ChIP-seq
☒	☐	Flow cytometry
☒	☐	MRI-based neuroimaging

Antibodies

Antibodies used	Goat anti-human IgG Fc antibody conjugated to HRP (Abcam, ab98624) and rabbit anti-human IgG and IgM antibody conjugated to HRP (Jackson ImmunoResearch, #309-035-107)
Validation	The goat anti-human IgG Fc antibody conjugated to HRP (Abcam, ab98624) was isolated from goat antisera by affinity chromatography using antigen coupled to agarose beads and conjugated to horse radish peroxidase. The antibody was tested by the manufacturer for suitability for use in ELISA and shown to have minimal crossreactivity with chicken, cow, goat, horse, mouse, rabbit, rat, and sheep antibodies.<br>The rabbit anti-human IgG and IgM antibody conjugated to HRP (Jackson ImmunoResearch, #309-035-107) was isolated from rabbit antisera using antigen coupled to agarose beads and conjugated to horse radish peroxidase. Based on immunoelectrophoresis and/or ELISA, the antibody reacts with both human IgG and IgM. It also reacts with the light chains of other human immunoglobulins. No antibody was detected against non-immunoglobulin serum proteins. The antibody was tested by the manufacturer for use in ELISA with minimal cross-reaction with mouse serum proteins, but it may cross-react with immunoglobulins from other species.

Human research participants

Policy information about studies involving human research participants

Population characteristics	Male and female patients, ages 21-70 (Phase 1a) or 21-75 (Phase 1b), with serum uric acid >6 mg/dL were screened for enrollment. Inclusion and exclusion criteria are provided in Supplemental Table 1 and patient demographics by cohort are provided in Supplemental Table 2 (Phase 1a) and Supplemental Table 4 (Phase 1b).
Recruitment	In the Phase 1a study, patients were recruited at four U.S. clinical research centers (Duncansville, PA, Dallas, TX, Miami, FL, and Orlando, FL). In the Phase 1b study, patients were recruited at eight U.S. clinical research centers (Little Rock, AR, Miami, FL, Orlando, FL, Baltimore, MD, Minneapolis, MN, Duncansville, PA, Anniston, AL, Lakewood, CO). These are large clinical trial research centers that recruit for many Phase 1 trials. There may be a selection bias for patients that volunteer for various clinical studies. Potential selection bias is unlikely to affect the key results of the clinical study because the primary bioanalytical endpoints, reduction in serum uric acid and prevention of anti-drug antibodies, are unlikely to be influenced by individual subjects. A phase 3 clinical trial of a similar recombinant pegylated uricase enzyme (pegloticase) was shown to be similarly effective in reducing serum uric acid after the initial dose in all subjects and the enzyme was similarly immunogenic in 92% of subjects (Sundy et al., JAMA. 2011;306(7):711-720). Moreover there was no placebo effect on serum uric acid levels noted in this trial.
Ethics oversight	The protocol and its amendments, the consent form, and other relevant study documentation were approved by Copernicus Group (Durham, NC), a central Institutional Review Board (IRB) and by each study center before initiation of the study.<br>This study was conducted in accordance with the ethical principles that have their origin in the Declaration of Helsinki and that are consistent with Good Clinical Practices and applicable regulatory requirements.<br>This statement is included in the manuscript

Note that full information on the approval of the study protocol must also be provided in the manuscript.

Clinical data

Policy information about clinical studies

All manuscripts should comply with the ICMJEguidelines for publication of clinical research and a completedCONSORT checklist must be included with all submissions.

Clinical trial registration	Phase 1a - NCT02464605; Phase 1b - NCT02648269
Study protocol	The Phase 1a and Phase 1b clinical trial protocols were provided as Supplemental Review Material
Data collection	In the Phase 1a study, patients were recruited at four U.S. clinical research centers (Duncansville, PA, Dallas, TX, Miami, FL, and Orlando, FL). In the Phase 1b study, patients were recruited at eight U.S. clinical research centers (Little Rock, AR, Miami, FL, Orlando, FL, Baltimore, MD, Minneapolis, MN, Duncansville, PA, Anniston, AL, Lakewood, CO). Phase 1a was initiated 01-May-2015 and completed 11-Nov-2015. Phase 1b was initiated 21-Dec-2015 and completed 28-Dec-2016.<br><br>This information has been added to the manuscript
Outcomes	The primary endpoint for both the Phase 1a and 1b studies, which involved experimental therapies, was safety and tolerability. Investigators at each site were responsible for recording all treatment-emergent adverse events (TEAEs). In addition to TEAESs, safety assessments included evaluation of clinical laboratory tests, 12-lead electrocardiogram (ECG), vital signs, physical examination and immunogenicity (IgG antibodies to SEL-037 and uricase, and IgM antibodies to polyethylene glycol [PEG]). The secondary endpoints included assessment of the activity of pegadricase as measured by serum uric acid levels and serum uricase activity and the immunogenicity of pegadricase, as measured by anti-uricase IgG antibodies and anti-PEG antibodies.
